# Fallen eyeball injury^☆^

**DOI:** 10.1016/j.bjorl.2017.04.008

**Published:** 2017-05-13

**Authors:** Seiji Hosokawa, Katsuyoshi Suzuki, Katsura Hakamada, Yasuhiro Hayashi, Hiroyuki Mineta

**Affiliations:** a Hamamatsu University School of Medicine, Department of Otorhinolaryngology Head & Neck Surgery, Hamamatsu, Japan; b Seirei Hamamatsu General Hospital, Department of Otorhinolaryngology, Hamamatsu, Japan; c Seirei Yokohama General Hospital, Department of Otorhinolaryngology, Yokohama, Japan

## Introduction

Orbital fractures are common and challenging to manage. They deserve special consideration because inappropriate management may result in an incorrect globe position and and/or compromised vision.^1^ In this rare case report, we describe a patient who sustained an ocular injury after falling on a toilet paper stand and was treated successfully with ophthalmic plastic and reconstructive surgery. To our knowledge, there have been no previous reports of orbital fracture with a fallen eyeball.

## Case report

**Figure 1:**
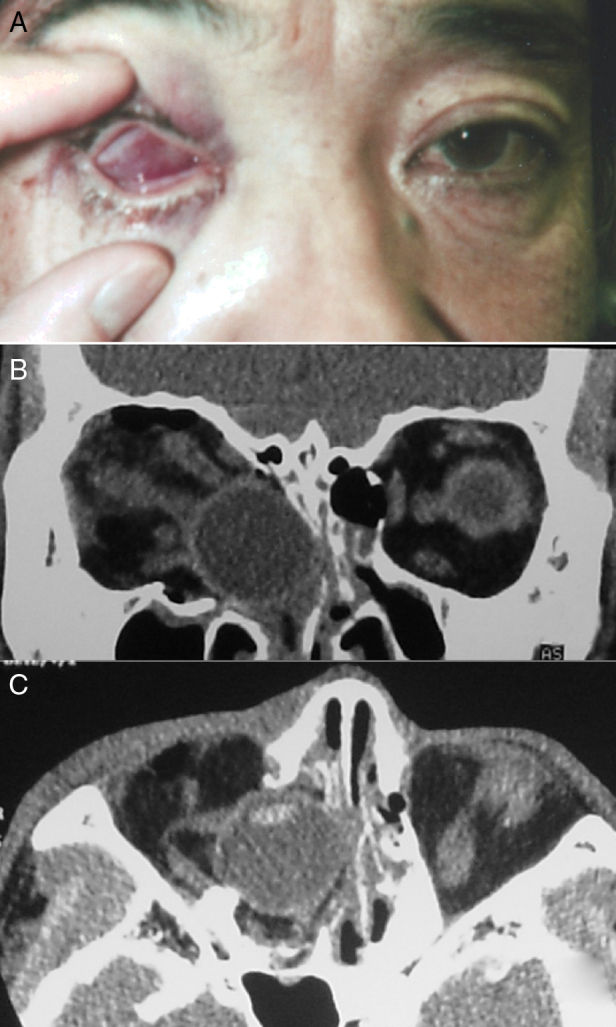
(A) Appearance of the right eye at initial presentation. (B) and (C) Fractures of the orbital floor and medial wall with a fallen globe were identified on computed tomography.

A 48 year-old man collapsed in his bathroom at home during an episode of unexplained dizziness. He fell forward and struck his right eye on a toilet paper stand. When he stood up, he noticed his right eye was inverted with dull pain and that he could not open his eyelids on that side. He visited his local emergency room and was referred to our hospital for further investigation. On examination, there were no signs of injury to the forehead or face. His right globe was inside the orbit and his eyelids were tightly closed (Fig. 1A). Vision and ocular motility could not be assessed. There was marked lid oedema on the right side. A computed tomography scan revealed that the bony orbit was fractured and that the globe had fallen into the ethmoid sinus; however, intracranial structures were unaffected and intact. There was no clear radiological evidence of right optic nerve avulsion. The anatomy of the extraocular muscle cone and fat was distorted on the right side (Fig. 1B–C). The other eye was clinically unremarkable.

**Figure 2:**
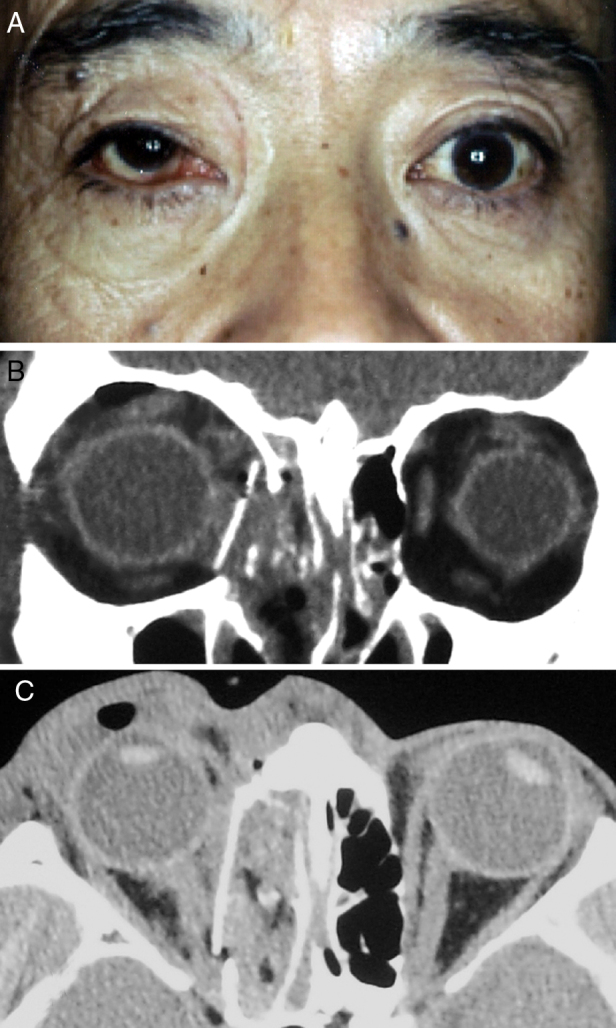
(A) The right eye after ophthalmic plastic and reconstructive surgery. (B) and (C) We constructed the inferomedial structures by inserting a preformed silicone plate on the orbital floor and medial wall.

On the following day, the patient underwent ophthalmic plastic and reconstructive surgery under general anaesthesia. The orbital floor was explored using a transconjunctival approach. When the orbital rim was reached, the dissection was continued in the subperiosteal plane into the orbit and then along the orbital floor. Deeper in the orbit, a malleable retractor was used to elevate the orbital tissues, making it easier to identify the orbital fracture defect. The orbital tissue was seen to be herniating into the ethmoid sinus. The orbital tissue was approached in a side-to-side fashion to avoid inadvertent penetration of the tissue with the elevator and to avoid exposure of the orbital fat, which would make visibility worse when attempting to retract displaced the eyeball. Segments of bone limiting elevation of soft tissue could be removed to improve mobility of the eyeball with the orbital tissue back into the orbit. The medial wall was accessed via a transcaruncular approach. The inferior and medial dissections were combined to create a single dissection plane and the prolapsed orbital soft tissues were returned to their correct positions. By introducing the periosteal elevator into the maxillary and ethmoidal sinus through the fractured bone defect, we were able to identify the margins of the fractured area and measure the extent of the defect. The site of the defect on the medial wall was covered by 3 mm thick porous polyethylene sheets cut into pieces and inserted into the ethmoidal sinus. The last step was to assess baseline ocular motility by a forced duction test. A fractured orbital wall can be repaired either by returning the existing bone to its original position or by replacing the wall with synthetic materials made especially for the orbit (Fig. 2A). The globe was moved forward and pulled up into place (Fig. 2B–C).

The patient was subsequently treated with methylprednisolone 1000 mg/day for 3 days. His postoperative course was uneventful. Following corticosteroid therapy, visual acuity in the affected eye improved from 6/60 to 6/7·5. Postoperative Goldmann perimetry showed a relative defect in the inferior nasal quadrant in the right eye. Ocular movements were entirely absent. Follow-up examination one year later showed no evidence of abnormal ocular movements but there was no improvement in visual acuity or the relative defect.

## Discussion

Traumatic eyeball displacement has a number of causes, the most common of which are motor vehicle and bicycle accidents, followed by falls.^2^, ^3^ Ocular injury caused by collision with a toilet paper stand is hardly common in everyday life and has never been reported as a cause of significant ocular or periocular injury. Complications associated with this type of injury include exposure keratitis, corneal abrasion, blurred vision, pain, blepharospasm, and anxiety. The incidence of optic nerve avulsion is also increased in patients who sustain traumatic eyeball displacement.^2^ Brain and orbital computed tomography scans are required to exclude intracranial bleeding, injury to the optic chiasm, and bone fractures. A fallen eyeball should be managed as an emergency because patients are at high risk of permanent loss of light perception.^3^ The naso-orbital-ethmoid complex is a delicate three-dimensional anatomical structure.^4^, ^5^ However, our patient did not suffer marked loss of visual acuity or visual field. How is the eye protected during such trauma? The eyeball (globe) lies protected in a bed of fat and connective tissue that is in turn contained inside a thin bony cone. Extraocular muscles attached to the globe assist movement of the globe in all directions. This arrangement protects the eye from severe trauma by transferring the force of the trauma first to the fat pads and then to the surrounding bony walls.

A specific surgical procedure is required to repair an orbital fracture accompanied by displacement of the eyeball. It is important to assess baseline ocular motility by a forced duction test. Damage to the eye, as occurred in our case, may cause severe dysfunction and disfigurement.^6^, ^7^ After the forced duction test, the tissues and eyeball can be mobilized into the orbit and separated from the orbital floor, medial bone fracture fragments, and the sinus mucosa. We used porous polyethylene sheets as implant materials that could be fixed to the orbital rim. This implant material is highly biocompatible, is easily trimmed into the required shape, has a low risk of infection, and has good strength and long-term stability. The implant can usually be removed easily if needed.^8^

In rare situations, as in our patient, trauma can also damage the globe itself or the nerve that connects the globe to the brain. This causes permanent loss of vision and requires immediate presentation to a hospital emergency room.

## Conclusion

We encountered a very rare case of a fallen eyeball injury caused by a fall onto a toilet paper stand. The patient was successfully treated with ophthalmic plastic and reconstructive surgery.

## Conflicts of interest

The authors declare no conflicts of interest.
